# Rapid and Serial Quantification of Adhesion Forces of Yeast and Mammalian Cells

**DOI:** 10.1371/journal.pone.0052712

**Published:** 2012-12-20

**Authors:** Eva Potthoff, Orane Guillaume-Gentil, Dario Ossola, Jérôme Polesel-Maris, Salomé LeibundGut-Landmann, Tomaso Zambelli, Julia A. Vorholt

**Affiliations:** 1 Institute of Microbiology, ETH Zurich, Zurich, Switzerland; 2 Institute for Biomedical Engineering, ETH Zurich, Zurich, Switzerland; 3 SPCSI Service de Physique et Chimie des Surfaces et Interfaces, CEA-Saclay Commissariat à l'énergie atomique, Gif-sur-Yvette, France; Laas-Cnrs, France

## Abstract

Cell adhesion to surfaces represents the basis for niche colonization and survival. Here we establish serial quantification of adhesion forces of different cell types using a single probe. The pace of single-cell force-spectroscopy was accelerated to up to 200 yeast and 20 mammalian cells per probe when replacing the conventional cell trapping cantilever chemistry of atomic force microscopy by underpressure immobilization with fluidic force microscopy (FluidFM). In consequence, statistically relevant data could be recorded in a rapid manner, the spectrum of examinable cells was enlarged, and the cell physiology preserved until approached for force spectroscopy. Adhesion forces of *Candida albicans* increased from below 4 up to 16 nN at 37°C on hydrophobic surfaces, whereas a Δ*hgc1*-mutant showed forces consistently below 4 nN. Monitoring adhesion of mammalian cells revealed mean adhesion forces of 600 nN of HeLa cells on fibronectin and were one order of magnitude higher than those observed for HEK cells.

## Introduction

The adhesion of microbial and mammalian cells to abiotic or biotic surfaces is mediated by a complex interplay of dynamically regulated specific and non-specific interactions. Microbial cell development, metabolic activity and cell viability are strongly affected by cell adhesion [1], which represents the initial step in biofilm formation. Mammalian cell adhesion is involved in cell differentiation, tissue development, inflammation, and infection and is thus highly important in the regulation of cell physiology. Although qualitative and semi-quantitative data on adhesion forces have been obtained using optical microscopy and flow-chamber approaches, the first set of quantitative data was generated using optical or magnetic tweezers and micropipettes [2]–[8]. Force spectroscopy approaches using atomic force microscopy (AFM) [9] have provided novel information on cell adhesion, particularly at the single-molecular level, and on the nanomechanical properties of cells [10]. Single-cell force spectroscopy (SCFS) of eukaryotic [11]–[13] and prokaryotic [14], [15] cells, which are attached to the end of a tipless AFM cantilever, allows the quantification of the adhesion force of the entire cell to a given substrate and makes possible the study of the contribution of distinct molecular classes to the overall adhesion event [10]. This force quantification helps unravel the adhesion-related gene products, mechanisms and regulatory signals [16]. State-of-the-art AFM measurements involve a chemical functionalization of the cantilever to irreversibly “glue” the cell of interest to the cantilever [10]. Consequently, each cell requires a separate cantilever that must be functionalized and calibrated, which impedes the ability to obtain high-throughput measurements. The process of the chemical fixation of cells is time consuming (typically 30 min [17]) and might alter the physiology of the cell, e.g., by affecting the cell surface [16], [18]. In addition, the cell must be amenable to chemical fixation, which is a procedure that often necessitates a preceding empirical optimization. The force range that can be recorded is limited by the force with which the cell is bound to the cantilever, which generally results in the application of only relatively short contact times between the cell and the substrate of less than one hour before the adhesion force exceeds the detectable range [18]. In this manuscript, we describe and apply a method that utilizes SCFS to enable the use of a single probe for the experimentation of multiple cells, which drastically reduces the time required to obtain statistically relevant data compared with conventional SCFS. In addition, the fixation of the cell to the cantilever, which occurs within a few seconds before the SCFS, allows for the force determination of cells in contact with the substrate for long periods of time. The method was validated using *Saccharomyces cerevisiae*, the most investigated eukaryotic model organism, and the dimorphic yeast *Candida albicans*, which is a well-studied human pathogen that presents a major threat to immunocompromised patients [19]; however, information on its adhesion forces is currently lacking. The mammalian cells used in this study comprise HeLa cells, the oldest and most commonly used standard immortal cancer cell line [20], and HEK cells, which were originally derived from human embryonic kidney cells. For the first time, we were able to show the use of serial SCFS experiments using a single cantilever for multiple mammalian cells. We performed SCFS experiments using a fluidic force microscope (FluidFM)-based method to investigate the adhesion of microbial and mammalian cells by performing serial, dynamic long-term adhesion measurements and demonstrated the universality and broad applicability of this method for different cell types with a wide force range.

## Results and Discussion

### Establishment of FluidFM-based single cell force spectroscopy (SCFS) using yeast cells

The principle of using micro-channeled probes that are directly connected to an external fluidic circuit that is operated by AFM (FluidFM) has been recently introduced and allows for the operation of this technique in liquid on top of an inverted optical microscope [21].

**Figure 1:**
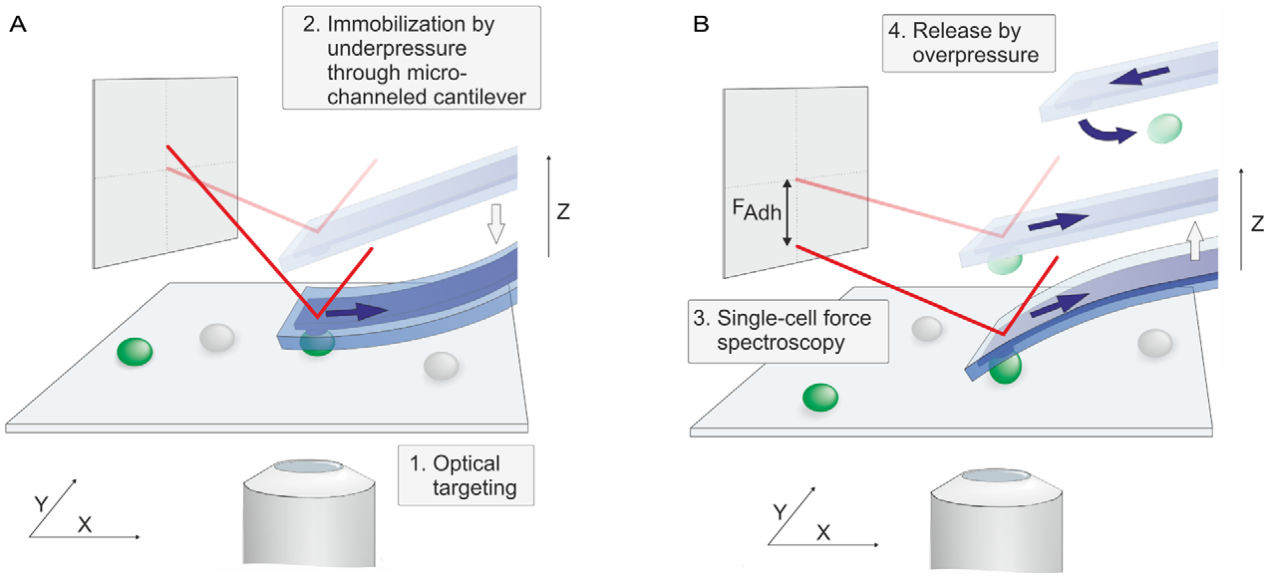
FluidFM-based single-cell force spectroscopy. Schematic view of the experimental principle. (A) Cell targeting and immobilization to the cantilever through the application of under pressure. (B) Single-cell force spectroscopy and subsequent release of the measured cell.

In this study, we adapted this technology to measure adhesion forces of individual cells. For SCFS, a cell is optically selected and subsequently approached in contact mode with a defined force set point (see Materials and Methods for details). During a pause of a few seconds with force feedback on, the cell is immobilized to the cantilever by applying underpressure through the micro-channeled probe (Figure 1A). The latter is kept constant, while the probe is then retracted to record the deflection signal from which the force required to detach the cell is deduced. Once the cell is detached from the substrate, an overpressure pulse expels the cell from the cantilever aperture such that another cell can be optically targeted and approached (Figure 1B). Thus instead of chemically immobilizing the cells to the cantilever as in conventional AFM experiments, we applied underpressure to fix the cell to the cantilever aperture [22]. Because cells are not attached to the cantilever from the beginning, as in conventional AFM experiments, a dedicated experimental protocol was established. This procedure required the proper integration and adjustment of the AFM, microfluidics and optics. To this end, a digital pressure controller was implemented into the system used and a dedicated software was written for the coordinated application of the under- and overpressure by the pressure controller during the force-spectroscopy routine, which is regulated by the AFM controller. In consequence, a defined under- and overpressure at the desired time point for a certain amount of time became possible, thereby ensuring the reproducibility of the adhesion experiments. The technical details of the procedure requirements and other key modifications to the AFM laser, the microfabrication of the cantilever, and the antifouling coating of the cantilever with poly (L-lysine)-grafted-poly (ethylene glycol) (PLL-*g*-PEG) are outlined in Materials and Methods.

**Figure 2:**
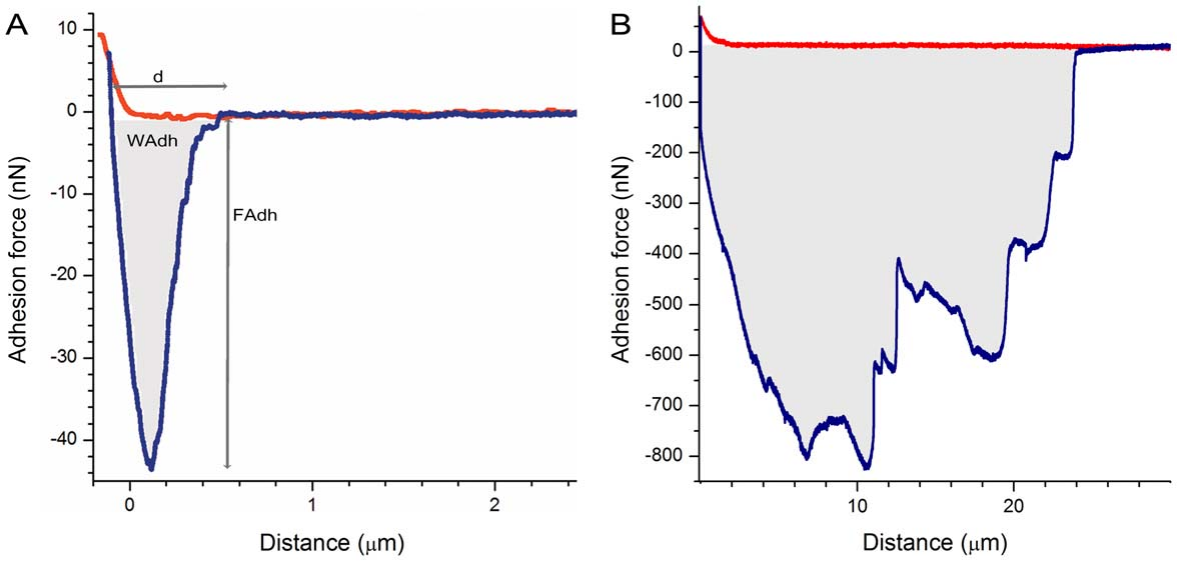
Representative example of the force-distance (F–D) curves that were obtained with a *C. albicans* cell on DDP (A) and a HeLa cell on fibronectin (B). The data show a force range between 20 and 800 nN (red: approach, blue: retraction curve). The maximal adhesion force was computed as the minimum force value (F_Adh_). The work performed by the Z-piezo during the detachment process (W_Adh_) was calculated as the area below the baseline (shaded area). The distance (d) is the distance required for the complete separation of the yeast cell from the substrate.

In a first set of experiments, *C. albicans* was used as the model organism. The force curves were recorded during the detachment of the cell, which was accomplished through the retraction of the cantilever. At the end of each SCFS, a short overpressure pulse released the attached cell, which allowed the cantilever to be immediately used again. This reutilization of the same hollow cantilever made possible the measurement of individual cells within a short time and the collection of up to 200 force curves for different yeast cells using a single cantilever in one day. The force spectroscopy routine required only a few minutes to target, immobilize, and release the cell as well as to change the cantilever position to the next cell. A representative force-distance curve that was obtained with *C. albicans* on a hydrophobic dodecyl phosphate (DDP) surface is shown in Figure 2A. This curve was used to extract the maximal adhesion force (F_Adh_), the adhesion work (W_Adh_), that was performed by the 10 µm Z-piezo and the distance (d) required to detach the cell completely from the substrate (in this case, F_Adh_  = 43 nN, d = 650 nm and W_Adh_  = 8×10^−15^J).

**Figure 3:**
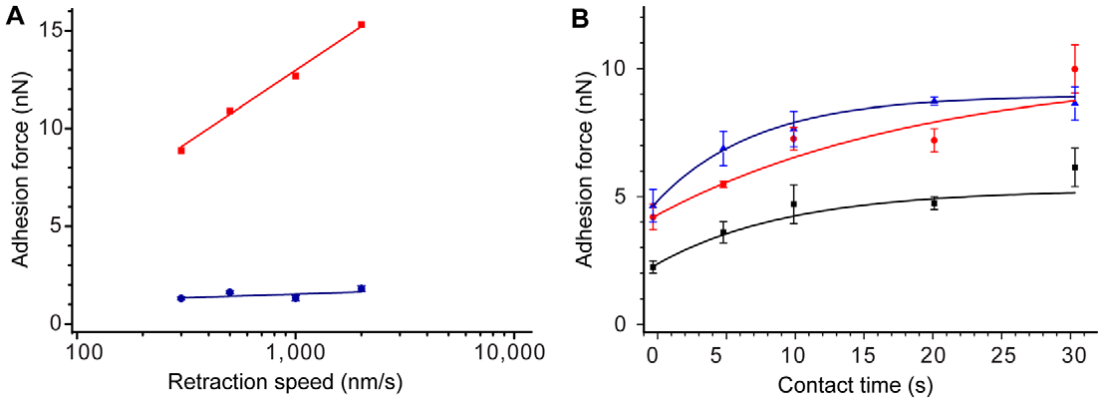
The adhesion forces depend on the retraction speed and the contact time. (A) Dependence of the adhesion force on the retraction speed with a constant contact time of 30 s. The data were obtained with a *C. albicans* cell on DDP (red) and glass (blue). (B) Dependence of the adhesion force on the contact time. The data were obtained using a *C. albicans* cell on DDP at three different retraction speeds: 300 nm/s (black), 500 nm/s (red) and 2000 nm/s (blue). Between 5 and 10 F–D curves were randomly recorded per condition. The data shown represent the mean ± standard error. The results in A and B demonstrate that repeated measurements with the same cell do not exhibit a high variance in the adhesion forces.

A convention procedure in standard SCFS is the verification of the dependence of the adhesion on the retraction speed and the contact time. Evans et al. showed how a general potential landscape is modified by the application of an external force and described dynamic effects of AFM force spectroscopy [23]. So far, the majority of dynamic force spectroscopy studies were carried out to assess the strength of individual bonds. However, retraction speed dependence was also observed with living cells, reflecting multiple binding/unbinding events [24]. Notably, we observed that also the increase in the adhesion force on a DDP-coated surface was correlated with the logarithm of the retraction speed (Figure 3A). A general quantitative model of hydrophobic forces is still object of intense theoretical investigations [25]–[28]; therefore, a direct interpretation of our data is still impeded. Furthermore, we observed the expected correlation of the adhesion force with increasing contact time of the cell with the surface (Figure 3B). In contrast to all other experiments described below, to validate the SCFS results by FluidFM in these experiments, we aspired individual yeast cells to the aperture before the cantilever with the attached cell was approached to the substrate. These experiments indicated that the chemical functionalization for the fixation of the cell to the cantilever that is performed in conventional SCFS experiments can be circumvented altogether. This step can instead be replaced by the physical sucking of the cells to the aperture of the hollow cantilever. The release of the cell by applying an overpressure pulse makes the immediate reutilization of the cantilever possible, thereby resulting in the ability to perform serial measurements.

### Dynamic adhesion forces of yeast cells at different temperatures

**Figure 4:**
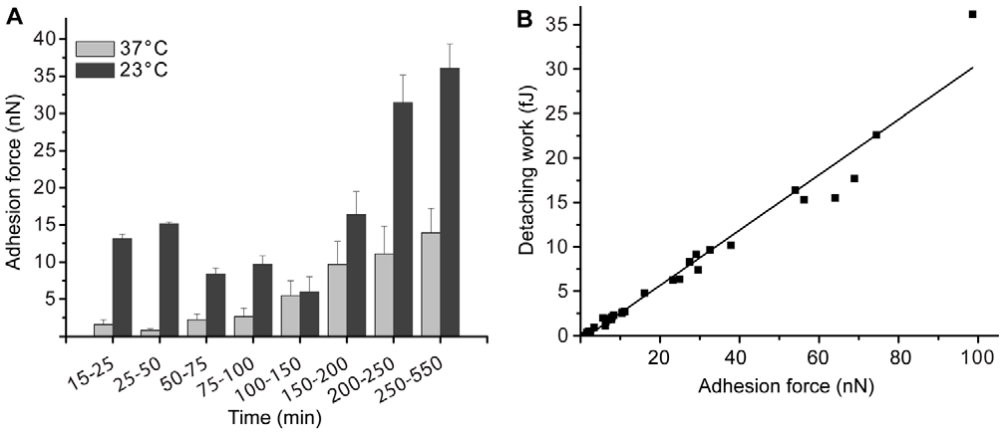
Adhesion of *C. albicans* to moderately hydrophobic substrates. (A) Time-dependent comparison of the maximal adhesion forces at 23 and 37°C. (B) Correlation of F_Adh_ and W_Adh_ at 23°C throughout the 9 hours of adhesion; R^2^  = 0.96. The analysis in (A) involved the recording of at least 7 F–D curves per condition and time frame. The data represent the mean ± standard error.

FluidFM-based SCFS leaves cells unaltered until the moment when the cell is targeted for approach and fixed to the cantilever by aspiration, which only takes a few seconds. Consequently, for the first time the dynamic behavior of cells during the adhesion process can be studied over the course of hours and principally days. We used *C. albicans* as a model organism because it is well established that this microorganism exhibits different cell surface properties when exposed to different environmental conditions [19]; yet, quantitative data reporting whole cell adhesion forces are currently lacking. The yeast form of *C. albicans* cells are more hydrophobic at room temperature compared with those cells grown at 37°C [29]. To correlate these findings with the adhesion forces, SCFS experiments were performed with *C. albicans* adsorbed on moderately hydrophobic surfaces (Materials and Methods) at varying adhesion times and temperatures. Indeed, longer incubation times on the surfaces resulted in higher adhesion forces and cells that were incubated at 23°C exhibited higher adhesion forces than those that were incubated at 37°C during the entire course of the experiment (9 hours, Figure 4A). Thus, the more hydrophobic yeasts, grown at 23°C, are interacting stronger with the moderate hydrophobic substrate compared to the more hydrophilic yeasts, grown at 37°C. A total of 141 cells were measured and thus reliable force data for the yeast population were obtained. The data demonstrated that the adhesion forces increased as a function of the incubation time and depended on the temperature. As expected and consistent with conventional SCFS, a linear correlation was observed between the measured adhesion forces (F_Adh_) and the performed work (W_Adh_), as shown in Figure 4B.

### Comparison of the adhesion forces of two yeast types to different substrates

**Figure 5:**
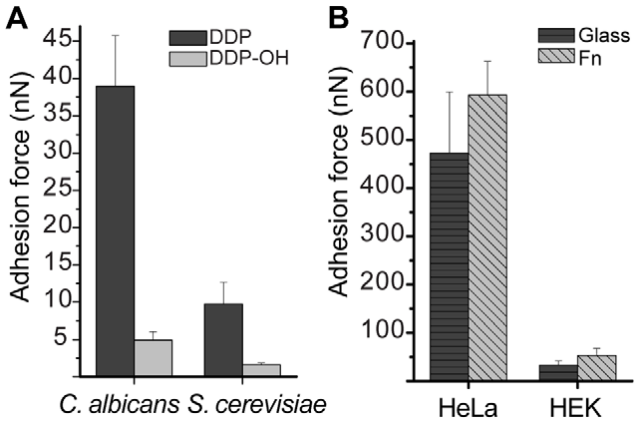
Maximal adhesion forces of yeast and mammalian cells to abiotic substrates. (A) Comparison of the maximal adhesion forces of *C. albicans* and *S. cerevisiae* to hydrophobic DDP and hydrophilic DDP-OH surfaces at 30°C after an adhesion time of 15 min. The data represent the mean ± standard error of 5–14 measurements per yeast and substrate. (B) Comparison of the maximal adhesion forces of HeLa and HEK cells to glass and fibronectin-coated substrates at 37°C after overnight contact with the cell substrate. A total of 12 and 11 HeLa cells were measured on the glass and fibronectin substrates, respectively, whereas 8 and 9 HEK cells were measured on the glass and on fibronectin substrates, respectively. The data represent the mean ± standard error. The force spectroscopy data, which were obtained when the cell was detached from the cantilever, were not included in the mean and error calculations.

After validating SCFS using FluidFM and demonstrating the utility of the method for rapid and serial measurements, we investigated the adhesion forces of different types of yeast cells to abiotic surfaces. Due to the hydrophobic nature of the surface [30], *C. albicans* is expected to adhere to a DDP-coated surface through non-specific hydrophobic interactions. The adhesion force of *C. albicans* to DDP was determined to be 39±7 nN at 30°C (Figure 2A and 5A, Table 1). A hydrophilic surface (DDP-OH, see Materials and Methods) was used as a control. As expected, the adhesion force was significantly reduced to 10±3 nN upon adhesion to the hydrophilic surface (Figure 5A, Table 1). To compare the adhesion forces of different yeast genera, we also studied the model yeast *S. cerevisiae* and found that its adhesion forces to the hydrophilic and hydrophobic surfaces were 2.0±0.3 nN and 5±1 nN, respectively (Figure 5A, Table 1).

**Table 1:** Comparison of the maximal adhesion forces of all cell types on different substrates.

Substrate	Cell type	Mean ± StEr (nN)
DDP	*C. albicans*	39±7
	*S. cerevisiae*	5±1
DDP-OH	*C. albicans*	10±3
	*S. cerevisiae*	2±0.3
Glass	HeLa	473±127
	HEK	33±9
Fibronectin	HeLa	593±70
	HEK	53±15

The same behavior, which involves a higher adhesion to hydrophobic surfaces than hydrophilic substrates, was obtained with a different *S. cerevisiae* strain (NCYC 1681, commonly used in ale breweries) through conventional AFM-based SCFS with chemical fixation of the yeast cells [12]. The differences in the absolute values can be explained by the different strains and experimental conditions. Consequently, the data show that we were able to, for the first time, demonstrate that fast, serial measurements of yeast cell adhesion can be obtained using FluidFM to monitor the adhesion forces to specific substrates and to quantify the long-term adhesion interactions. The comparison of two different yeast genera demonstrated that the method is generally applicable to these types of otherwise well-studied microorganisms.

### Comparison of the adhesion forces of wild type and mutant yeast cells on an identical substrate

**Figure 6:**
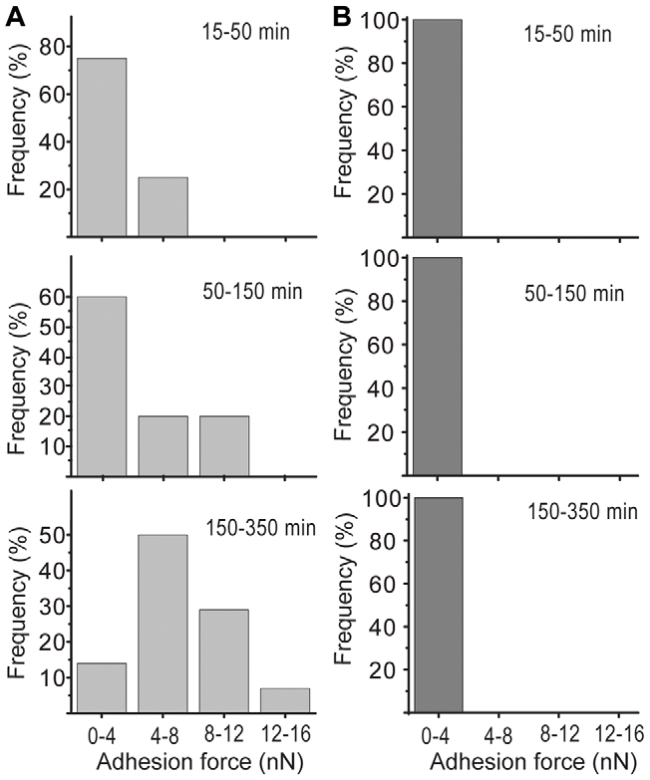
Comparison of adhesion forces of *C. albicans* wild type and Δ*hgc1* cells at 37°C. (A) Distribution of adhesion forces of *C. albicans* wild type and (B) Δ*hgc1* cells on moderate hydrophobic substrate. Yeasts were grown at the same temperature the experiment was performed.

In further experiments we applied the established method to investigate changes in *C. albicans* surface hydrophobicity depending on the morphological appearance. *C. albicans*, a dimorphic yeast, can exist as yeast cell or in a hyphal form. While we focused on the yeast form in the above described experiments, we noticed an elevated fraction of yeast cells that produced hyphae upon adhesion at 37°C to a solid surface in phosphate-buffered saline (PBS) (from ∼5% of the cells in the pre-culture and at the beginning of the experiment to ∼40% after 5 hours of incubation on the solid surface). In contrast, no hyphal formation was observed at 23°C, which is in agreement with the existing literature [31]. HGC1 has been identified as an essential G1, cyclin-related protein for hyphal morphogenesis [32]. Although the morphological transition from yeast to hyphal form had previously been characterized [31], it was not known whether a Δ*hgc1* mutant would exhibit changes in its surface hydrophobicity and, consequently, altered interaction properties with hydrophobic substrates. We compared the adhesion behavior of *C. albicans* wild-type cells and Δ*hgc1* mutants at 37°C over time. To ensure that both cell types were tested on an identical surface, we used a wild-type strain expressing green fluorescent protein (GFP) and an unlabeled Δ*hgc1* mutant. To distinguish both strains we took advantage of the combination of the FluidFM and fluorescence microscopy. As assessed in the previous experiment, the *C. albicans* wild-type cells underwent a shift from low (1.3±0.4 nN) to high (15.6±2.5 nN) adhesion forces as a function of incubation time on the solid, moderate hydrophobic (Materials and Methods) surface (Figure 6A). In contrast, the Δ*hgc1* mutant remained at a low force level of 0.7±0.2 nN throughout the entire experiment (Figure 6B).

These findings suggest that a defect in *hgc1* not only arrests *C. albicans* in the yeast form [31] but also prevents the changes in cell surface hydrophobicity that normally occur at 37°C during cell adhesion to moderate hydrophobic surfaces.

### The use of FluidFM-based SCFS to measure the adhesion of mammalian cells

The next goal of this study was to broaden the applications to measure the adhesion forces of mammalian cells. The adhesion of mammalian cells represents the sum of all adhesive interactions between the substrate and the cell [33] and is generally higher than the forces determined for yeast cells. In addition, several additional challenges needed to be tackled. While yeast cells are symmetric, spherical and quite stiff [34], [35], mammalian cells are more elastic and may spread more widely on the surface [36]. Thus, unwanted adhesion to the cantilever was expected that may render the serial measurements of mammalian cells more difficult compared with yeast cells. To date, few conventional AFM measurements with mammalian cells have been achieved. As one these Hosseini *et al*. could successfully demonstrate the quantification of time dependent cell-cell interaction forces applying AFM; however, at the same time the authors noted that SCFS is prone to fail at longer contact times because the adhesion forces between the cell and the substrate surpass those used for the fixation of the cells to the cantilever [37]. Consequently, the cells are brought in contact with the substrate for only one hour or less. Weder *et al*. used specific interactions through a fibronectin-coated cantilever to increase the binding strength to the cantilever and thus increased the time that the mammalian cells were adhered to the surface to 24 hours [17], [38]. Notably, the cell fixation to the fibronectin-coated cantilever required 30 minutes of contact [38], which is problematic. Mammalian cells recognize the fibronectin as an adhesion substrate, respond to the recognition by altering its gene expression and start spreading (on the cantilever surface) by specifically binding to integrins and transmembrane adhesion receptors, thereby affecting the molecular surface structure of the cell [4], [39]. We tested whether FluidFM can be used to detach mammalian cells from standard surfaces such that a long contact time between the cell and the substrate can potentially be used but only a minimal time is required for the cell fixation to the cantilever. We used mammalian cells that were incubated overnight under cell culture conditions to allow for cell spreading and the renewal of adhesion molecules on the substrate under physiological conditions. Up to this point, no chemical or mechanical force that might have led to additional intracellular rearrangements has acted on the cell of interest. Because mammalian cells are bigger (and more elastic) than yeast cells [34]–[36], a larger detaching distance is required [40]. In preliminary experiments, Dörig *et al*. used a 100 µm stepper motor to detach one cell and therewith precluding the acquisition of the force-distance curve because of the superimposed sinusoidal noise [22]. Here we used a 100 µm Z-piezo stage, which drastically reduced the noise level. We also used cantilevers with larger openings, i.e., 8 µm, compared to the 2 µm openings that we used with yeast cells. A representative force-distance curve that was recorded with a HeLa cell on fibronectin is shown in Figure 2B. Notably, we measured an adhesion force that was 40-fold higher (831 nN), a detachment distance that was approximately 35-fold longer (24 um), a higher adhesion work (1,7×10^−11^ J) and multiple detachment events, which are typically referred to as jumps or tethers [33], [41], compared to yeast cells. These results indicate the principle differences in the detachment of microbial and mammalian cells. Whereas the detachment of yeast cells occurs in roughly one large step (Figure 2A), the mammalian cell detachment involves a number of distinct unbinding events [41]. The mean overall adhesion force of HeLa cells to uncoated glass substrate was determined to be 470±130 nN at 37°C (Figure 5B, Table 1). In this experiment, twelve cells were measured in series using the same cantilever, which reduced the experimental time and the consumables. In spite of the anti-fouling coating of the cantilever with PLL-g-PEG, which is often referred to as one of the most efficient coatings that are currently available [42], the sticking of the mammalian cells could not be entirely prevented. Hence, it was not possible to obtain the same quantity of serial measurements that were obtained with yeast cells; however, we still were able to record, on average, 10 times more force curves than with conventional SCFS. The relatively high standard error demonstrates the high biological variability, which emphasizes the need to record a large number of force spectroscopies for the individual cells.

### Comparison of the adhesion forces of two mammalian cell types to different substrates

We then tested whether FluidFM-based SCFS could be used to measure stronger adhesion forces of HeLa cells. We therefore used fibronectin as the substrate instead of for the fixation to the cantilever as in the previous experiments [38]. As expected, the mean adhesion forces increased relative to those measured with the glass surfaces and were in the range of 590±70 nN (Figure 5B, Table 1). On occasion, the adhesion force between the cell and the fibronectin substrate surpassed the mechanical limit that is given by the maximal applied underpressure and the aperture area. In general, adhesion forces of up to 1600 nN were measured, which demonstrates the suitability of FluidFM for the measurement of the high adhesion forces of mammalian cells, which could not be measured with previous approaches. We then compared the HeLa adhesion forces to glass and fibronectin with a second cell line, i.e., HEK cells. The determined mean adhesion forces of HEK cells to glass and fibronectin were 33±9 nN and 53±15 nN, respectively (Figure 5B, Table 1). However, HeLa cells adhered more strongly to both substrates compared to HEK cells. To the best of our knowledge, these results demonstrate for the first time the serial, long-term measurements of the adhesion forces of these cell types, which show the dependency of these adhesion forces on the cell type and the substrate. Moreover, the cell fixation by underpressure allows the measurement of high forces after long contact times between the cell and the substrate, which ensures the unhampered cell spreading onto the substrate.

## Conclusions

The use of FluidFM-based SCFS opens new avenues for the acquisition of AFM-based adhesion force measurements. We used different microbial and mammalian cell types to show that this method is universally applicable to living cells and allows the measurement of the adhesion forces of cells for which conventional AFM approaches have so far been unsuccessful. The measured adhesion forces are in the range of 500 pN to 1600 nN, which is relevant for the measurement of the cell adhesion forces of mammalian cells and microorganisms, such as yeast. The upper physical limit of this range is restricted by the aspiration force required for the cell fixation process. However, this range increases the maximal measurable adhesion force by approximately one order of magnitude compared to conventional AFM approaches [41], allowing for quantification of long-term adhesion measurements to substrates which were not possible before. The hydrodynamic cell fixation to the cantilever circumvents the need to chemically modify the cantilever surface and minimizes the impact on the cell physiology. The cell fixation is performed a few seconds before SCFS. This short time allows the possibility of cell adaptation to the substrate prior to the SCFS measurements, which is particularly useful with mammalian cells. The serial SCFS procedure described in this manuscript not only permits the measurement of quantitative and statistically relevant data in a rapid manner but also makes it possible to monitor the temporal changes in long-term adhesion processes that are relevant for biofilm or tissue formation. The cell release after SCFS allows to obtain independent cell adhesion measurements, thereby resulting in a higher reliability in the recorded forces under the given conditions rather than the repeated measurement of individual cells, which may easily lead to artifacts [18]. Compared to conventional SCFS, the use of FluidFM-based SCFS makes it possible to conduct up to tenfold as many experiments per day (up to 200 experiments with FluidFM compared to approximately 20 with the traditional cantilevers). In conclusion, we were able to overcome a major obstacle in the development of high-throughput SCFS by developing a method that obtains sufficient statistics to make relevant biological statements within an acceptable time frame.

## Materials and Methods

### Yeast culture conditions

Wild-type *Candida albicans* (SC5314), wild-type expressing GFP (pACT1-GFP) [43], *C. albicans* Δ*hgc1* (WYZ12.2) [32] and wild-type *Saccharomyces cerevisiae* (W303) were grown in yeast extract-peptone-dextrose medium (10 g yeast extract (Oxoid), 20 g bacto peptone (BD) and 20 g glucose (Fluka) in 1 L ddH_2_O). The cultures (20 ml) were inoculated with an overnight pre-culture to obtain a cell number of 1×10^6^ cells/ml (determined by optical density) and grown for 15 hours at 180 rpm and a temperature of 23°C, 30°C or 37°C. Prior to the experiments, the cultures were washed three times as follows: a 1-ml aliquot of the culture was pelleted at 12,500 rpm (Eppendorf centrifuge 5424) for 1.5 min at room temperature and subsequently resuspended in 1 ml of filtered (0.22 μm pore size) phosphate-buffered saline (PBS: 8 g NaCl (Merck), 0.2 g KCl, 1.44 g Na_2_HPO_4_ and 0.24 g KH_2_PO_4_ (all from Fluka) in 1 L of distilled water, pH 7.4) [44]. The cell number was then adjusted to obtain approximately 50 cells/mm^2^ substrate in 5 ml PBS.

### Mammalian cell culture conditions

HeLa (ATCC) and HEK (generously provided by H. Abriel) cells were maintained in growth medium consisting of Dulbecco's modified eagle's media (DMEM, Invitrogen, Switzerland) supplemented with 10% fetal bovine serum (FBS, Chemie Brunschwig, Switzerland) and 1% penicillin/streptomycin (pen/strep, Invitrogen, Switzerland) at 37°C in a humidified atmosphere with 5% CO_2_. The incubation of the cell cultures in 0.25% trypsin-EDTA (Invitrogen, Switzerland) (HeLa cells for 2 minutes, HEK cells for 30 seconds) detached the cells from the culture dish, which allowed for the seeding of the cells onto the desired substrate (glass or fibronectin-coated glass). The cells were incubated overnight under the above-mentioned cell culture conditions to allow for cell spreading. Prior to the adhesion force measurements, the cells were washed three times with a filtered CO_2_-independent medium (Invitrogen, Switzerland) supplemented with 10% FBS (Invitrogen, Switzerland), 1% pen/strep (Invitrogen, Switzerland) and 2 mM L-glutamine (Sigma-Aldrich, Switzerland). The cell concentration was adjusted to obtain sufficiently high numbers of single cells on the desired substrate at the start of the experiment.

### FluidFM setup required for single-cell force spectroscopy (SCFS)

#### Digital pressure controller

To perform reproducible adhesion experiments, it is essential to maintain the influence on the cell of interest as small as possible. It is therefore crucial to use a defined pressure. In previous approaches, the system was previously operated manually with a 10-ml syringe [21], [22]; accordingly, only a rough estimation of the pressure in the microchannel was possible. In this study, we connected the microfluidic tubing system to a digital pressure controller (Cytosurge AG, Switzerland), which allowed the application of a defined under- and overpressure at a desired time point for a certain period of time. The range of an underpressure of 800 mbar to an overpressure of 1000 mbar allowed maximal aspiration forces for cell fixation of 250 nN and 3.5 µN with a 2 μm and an 8 μm cantilever, respectively. A force spectroscopy routine was developed exclusively for the FluidFM-based SCFS, which was synchronized with the microfluidic control. This synchronization qualified the setup for serial force measurements. Because cells are not attached to the cantilever from the beginning of the experiment as in conventional AFM experiments, the developed force spectroscopy routine included a forward and a backward force spectroscopy, which was discontinued by a pause for the hydrostatic immobilization of the cell (see below).

#### AFM laser/optical detection

An appropriate low background level (∼100 pN with the used cantilever) and a straight baseline are mandatory to obtain an accurate adhesion measurement. An electric laser control circuit was integrated with an amended modulation and in addition, the software and filter system, as well as the laser wavelength (860 nm), were optimized for best optical detection. Moreover, the cantilevers were coated with a gold layer to simultaneously obtain greater reflection and enable optical targeting through their transparency.

#### Optical filter

The ability to work with fluorescently labeled microorganisms represents a major advantage, especially in the analysis of smaller microorganisms and in the differentiation between two strains on the same surface. This ability helps reduce the number of control experiments and allows for gene expression studies as a consequence of the application of forces to the cell. However, the strong excitation light that is used for fluorescence microscopy causes laser disturbance. Therefore, a Hoya IR-76 infrared filter (Edmund Optics, UK) was incorporated in front of the detector in the AFM head. This filter permitted only the infrared (laser) light to pass and blocked all of the visible light. The laser itself was filtered from the light microscopy pathways using an infrared short-pass filter (800 nm, NT64-333 from Edmund Optics, UK).

#### Cantilevers

To adapt the cantilever sensitivity to the force range of interest, micro-channeled cantilevers were fabricated in different lengths after overcoming the challenges in their microfabrication, which were related to the robust attachment of the cantilever to the chip and to the complete etching of the sacrificial layer. The cantilever lengths from 100 to 350 μm correspond to stiffness values from 8 to 0.4 N/m. An optimal stiffness can be chosen with respect to the biological object and its adhesion properties (in this case, ∼2–3 N/m). The force detection range of the FluidFM system, which is based on the spring constant and the detection range of the AFM position sensor for the chosen cantilevers, is between 100 pN and 4 µN. Furthermore, pillars were added in the middle of the microchannel to facilitate the filling of the cantilever and ensure that this process occurred without the generation of any air bubbles.

#### Surface coating

The substrates, which presented a defined surface hydrophobicity, were generated as previously described [45]. Briefly, glass wafers coated with 50 nm-thick indium tin oxide (ITO) (MicroVacuum, Hungary) served as the substrate for a dodecyl phosphate (DDP)/hydroxy-DDP (DDP-OH) coating. The substrate was sonicated in ultra-pure water and 2-propanol (Scharlau, Spain) for 10 min at room temperature in a Branson 2210 Ultrasound bath, dried under flowing nitrogen gas and plasma cleaned (Plasma Cleaner PDG-32G, Harrick Plasma, USA) for 2 min immediately prior to the coating procedure. A 0.5 mM DDP solution (SuSoS AG, Switzerland) was prepared in filtered ultra-pure water. In the preparation of the hydrophobic substrates, the ITO substrates were immersed in 100% DDP for 19 h, rinsed with ultra-pure water and dried with nitrogen. The static contact angle measurements were performed on a Ramé-Hart contact angle goniometer on the freshly prepared surfaces with ultra-pure water drops of 6–8 μL. The contact angles of the hydrophobic substrates were determined to be between 100 and 110°. The same procedure was performed to generate the hydrophilic substrates; instead of DDP, however, a solution of 100% 0.5 mM DDP-OH in filtered ddH_2_O was used, which resulted in contact angles of approximately 45–55°. To obtain moderate hydrophobicity, the solutions were mixed at a ratio of 40∶60 vol % (DDP:DDP-OH), which resulted in contact angles in the range of 70–80°. For the preparation of the fibronectin-coated substrates, 50-mm glass dishes (WillCo Wells B.V., The Netherlands) were washed with 2-propanol (Scharlau, Spain), dried under flowing nitrogen gas and plasma cleaned (Plasma Cleaner PDG-32G, Harrick Plasma, USA) for 2 min immediately prior to the coating procedure. A 0.05 mg/ml fibronectin solution (Sigma-Aldrich, Switzerland) was prepared in filtered PBS. The glass dishes were emerged in the fibronectin solution for 45 minutes and subsequently rinsed with filtered PBS. In the investigation of the yeast-glass and mammalian cell-glass interactions, 50-mm dishes (WillCo Wells B.V., Netherlands) were used, after cleaning with 2-propanol (Scharlau, Spain) and drying with nitrogen.

### Cantilever preparation and calibration

Rectangular, tip-less, 150-μm-long silicon nitride probes with a 2-μm or an 8-μm aperture were chosen for the study of yeast or mammalian cells, respectively [22] (Cytosurge AG, Switzerland). The corresponding stiffness of ∼2.5 N/m was appropriate for the explored force range. The aperture was large enough to apply enough force with the given under-pressure to detach the cells from the substrate but small enough to obtain a tight seal. The cantilevers were plasma-cleaned prior to the deposition of an anti-fouling coating of 0.5 mg/ml PLL (20 kDa) that was *grafted* with PEG (2 kDa) (PLL-*g*-PEG) (Surface Solution SuSoS AG, Switzerland) in filtered ultra-pure water. PLL-*g*-PEG was used to diminish unspecific binding of the cells to the cantilever and was previously shown not to affect cell viability [22], [46]. The PLL-*g*-PEG solution was filled into a reservoir and, with an overpressure of Δ50 mbar, was pressed through the cantilever until it reached the aperture. The probe was simultaneously immersed in the same PLL-*g*-PEG solution to coat its exterior for 1 h and subsequently washed in filtered ddH_2_O for 5 min [47]. Prior to each experiment, the cantilever sensitivity was calibrated using software-implemented scripts based on the formalism described by Sader *et al*. [48] and was determined to exhibit a spring constant in the range of 1.9–2.7 N/m.

### SCFS procedures using FluidFM

A FluidFM (Cytosurge AG, Zürich and Nanosurf AG, Liestal, Switzerland) mounted on top of an Axio Observer D1 inverted microscope (Carl Zeiss, Jena, Germany) was used for the SCFS measurements. The FluidFM features a 10-μm Z-range linearized piezoelectric ceramic scanner and the previously described infrared laser. In addition, the FluidFM head was positioned on a 100-μm piezoelectric Z-stage fixed to the optical microscope for the mammalian cell experiments. The force measurements were recorded at 23, 30 or 37°C in an incubation chamber. Unless otherwise stated, a selected yeast cell was approached in contact mode with a set point of 10 nN. This approach was followed by a pause of 5 seconds with force feedback to apply the underpressure necessary (350 mbar in this study) to grasp the yeast cell. The mammalian cells were approached with a set point of 50 nN, which was followed by a pause of 3 seconds during which an underpressure of 700 mbar was applied. The probe was then retracted at a given piezo velocity to record the deflection signal (i.e., force); the specified underpressure was maintained during this process. Unless otherwise specified, an approach and retraction speed of 1 μm/s was applied over a pulling range of 9 μm for yeast and 60 μm for mammalian cells. Once the retracted position was reached, the underpressure was maintained for a few seconds to optically control whether the cell was detached from the substrate. An overpressure pulse of 1000 mbar was then applied to expel the cell such that the following cell could be optically targeted and approached. Before starting the adhesion measurements, the yeast cells were allowed to sediment and adhere to the substrate for 15 minutes, whereas the mammalian cells were kept on the desired substrate overnight under cell culture conditions. In the standard experiment, one force-distance (F–d) curve was recorded per cell. In the experiments that examined the dependency of the adhesion force on the retraction speed and the contact time, an individual yeast cell was aspirated to the aperture directly from the solution and used throughout the experiment.
